# The Impact of COVID-19 on Pediatric Malignancy Diagnosis and Treatment: Never the Same but Lessons Learned

**DOI:** 10.3390/vaccines11030667

**Published:** 2023-03-15

**Authors:** Ghadir K. Katato, Prasiksha Sitaula, Avanti Gupte, Eman T. Al-Antary

**Affiliations:** 1 Division of Hematology/Oncology, Children’s Hospital of Michigan, Detroit, MI 48201, USA; 2 Department of Pediatrics, Central Michigan University College of Medicine, Mt Clemons, MI 48603, USA; 3 Pediatric Blood and Marrow Transplantation Program, Division of Hematology/Oncology, Barbara Ann Karmanos Cancer Center, Children’s Hospital of Michigan, Detroit, MI 48201, USA

**Keywords:** COVID-19, oncology, pediatric, epidemiology, management, diagnosis

## Abstract

The severe acute respiratory syndrome coronavirus 2 (SARS-CoV-2) pandemic affected the pediatric oncology population globally. Over the course of 2 years, increasing reports have been made to better understand this entity and its pathologic complications on these patients. The pandemic has allowed healthcare providers, hospital systems, and leading oncologic societies to quickly adapt and formulate new guidelines for the effective understanding, management, and treatment of patients with pediatric malignancy.

## 1. Introduction

The outbreak of novel severe acute respiratory syndrome coronavirus 2 (SARS-CoV-2) first emerged in Wuhan and soon thereafter was declared by the World Health Organization (WHO) as a global pandemic [1]. The coronavirus disease 2019 (COVID-19) has caused tremendous strain on the healthcare systems worldwide and has captivated the interest of pediatric oncologists in a unique way. Adults with COVID-19 were reported to have a severe course of disease with a significantly higher mortality rate [2]. However, we now have data to show that most of the children who tested positive for COVID-19 had either asymptomatic infection or mild symptoms, with only a handful presenting with severe disease requiring critical care [3]. A similar trend has also been noted in our pediatric oncology patients compared to their adult counterparts [2,4]. The COVID-19 pandemic has posed significant challenges in the healthcare sector, which has directly or indirectly impacted the care of pediatric cancer patients. From the challenges of diagnostic delay and delay in hospitalization to interrupted treatment in COVID-19-affected oncological patients, there have been substantial obstacles faced by providers and patients during the pandemic [5,6].

This review aims to expand the existing knowledge base on COVID-19 in pediatric oncology patients by underlining some key causes and consequences of delay in diagnosis and interruption to cancer therapies, review recommendations provided by leading oncologic societies and highlight the management strategies adopted nationally and internationally during COVID-19 pandemic.

## 2. Materials and Methods

We reviewed a PubMed and Google scholar database search using the terms “COVID-19”, “Pediatric”, “Oncology”, “Malignancy”, “Cancer”, “Treatment”, “Management”, and “Diagnosis”. We applied peer-reviewed journals, human studies, and the English language to the filter search. We performed additional searches to identify articles related to topics of each discussion section. We read all identified articles completely, extracting and summarizing relevant information using our own language. We made the study selection based on prioritization and the strength of the type of study (prospective over retrospective), population size, and date of publication.

## 3. Epidemiology of SARS-CoV-2 in Pediatric Oncology

Over 15 million total COVID-19 childhood cases have been reported as of November 2022, which represents 18.3% of all the cases reported to date [7]. Per data published by the United Nations International Children’s Emergency Fund (UNICEF) on Nov 2022, over 16 thousand children have succumbed to COVID-19 and its associated complications [7]. These numbers might look alarming, but fortunately, it is only a small subset when compared to the adult population with COVID-19 [2]. COVID-19 in children caused overall less mortality and morbidity compared to the adult population [8]. When looking at children with cancer specifically, the deaths attributed to COVID-19 among pediatric oncology patients were 4% as per the global registry of COVID-19 in childhood cancer. The rate is much lower than the 13% reported in adult cancer patients [2].

Most of the cases of COVID-19 in pediatric cancer patients are asymptomatic or have mild to moderate symptoms. The Italian Association of Pediatric Hematology Oncology (AIEOP) analyzed data on the impact of SARS-CoV-2 among pediatric oncology patients during the second and third pandemic waves. These data showed that only a handful of patients had a severe or critical illness from the virus. More importantly, this report showed that infections occurring early on in the oncologic diagnosis had a worse outcome as compared to those infections occurring later on during the maintenance phase suggesting the importance of continuing chemotherapy, especially in those children on maintenance therapy [9]. In a systematic review by Schlage et al. that included 45 articles with over one thousand pediatric oncology patients with COVID-19, 41.7% had mild or moderate symptoms, 11.1% of patients showed severe COVID-19 symptoms, and 2.9% of patients with COVID-19 died, potentially due to complications related to COVID-19 [4]. Mukadda et al. carried out a cohort study comprised of children and adolescents with cancer across 131 institutions spanning over 45 countries. This study showed that a fifth of patients in this population had worse outcomes, especially those from lower-income countries, those with low absolute lymphocyte count (ALC) and absolute neutrophil count, and those who received intense therapy [10]. This suggests that increased mortality in resource-poor areas versus high-income areas was influenced by the availability of health resources and the ability to receive treatment in a timely manner.

In terms of new diagnoses, there are data suggesting a decreasing trend in new diagnoses of solid tumors compared to hematological malignancies during the pandemic era. This could possibly be explained by the acute nature of the presentation of the hematological malignancies, compelling patients to seek timely medical attention as opposed to the gradual presentation of most solid tumors [6]. However, it is interesting to note that the risk of COVID-19-related hospitalization has remained uniform in patients with solid tumors and hematologic malignancies [11].

Despite all of the available data, the gap still exists in understanding the immune-related response of COVID-19, especially among immunologically vulnerable pediatric cancer populations, and the lack of consistency in treatment strategies adopted to manage them.

## 4. Delay in Diagnosis of Pediatric Oncology Patients during the COVID-19 Pandemic

When COVID-19 first emerged as a global pandemic in 2020, it imposed a significant diagnostic dilemma and therapeutic challenges to already overwhelmed healthcare systems across the globe. Looking back at our experiences with previous pandemics, this appears to be a recurring problem. An overwhelmed healthcare system, when exposed to a novel and evolving disease for many reasons, fails to cope with the challenges associated with it and, unfortunately, affects the diagnosis of other diseases [5]. In pediatric oncology patients, where timely diagnosis and initiation of therapeutic modalities remain extremely crucial, these shortcomings in the system lead to significant delays in diagnosis and subsequent interruptions in their treatment [12,13,14]. Challenges, including overwhelmed health system, apprehension among parents and providers alike, staff shortages, lack of equipment, and lack of access to medication and supportive care, have made the oncology population more vulnerable during these trying times [15].

The direct impact of COVID-19 has been observed in the diagnosis of oncological cases, where the number of newly diagnosed cases has declined. Quarello et al. performed a retrospective analysis in a cohort in Italy and reported that there was approximately a 20.8% reduction in new diagnoses of pediatric cancer in the first lockdown period (March–May 2020) compared to the same time window since 2015–2019 [16]. A parallel finding by Chiaravalli et al. showed that only 45.7% of expected cases were registered in the lockdown period of COVID-19 compared to the same period in 2017, 2018, and 2019 [17]. Additional analysis by the same group showed that the observed cases in the COVID-19 period were local to the oncology center region location compared to cases registered from wider regions of Italy [17]. A similar trend of decreased number of new diagnoses and higher mortality within the peak of COVID-19 also holds true in other countries, including the United States (U.S.) [18]. In a study reported by Ding et al., delayed diagnosis was shown to be associated with more severe disease and a higher mortality rate [6]. These studies elucidate the impact of COVID-19 on the diagnosis of cancer in pediatric patients and shed light on some key causes and consequences of such delays.

One of the causes of delayed diagnosis is the reluctance to present to care. Apprehension among patients and providers alike has been described as the leading cause behind the reluctance to seek medical care in a timely fashion. COVID-19 has increased the fear of infection among patients. Several case studies of parents keeping children at home longer than usual before seeking care have been reported. In this study by Ciachhini et al., a 76% reduction in the number of admissions was reported for the emergency department in a children’s hospital, and the most significant reduction was associated with patients admitted with trivial issues [19]. This fear of infection, coupled with guidelines put in place by the government for the effective lockdown to maintain a low infectivity rate, has caused patients and their families to become less inclined to seek medical help unless in cases of emergency. There have been instances where even after the diagnosis, the patient has failed to maintain regular follow-up appointments or lab checkups due to fear of infection, which ultimately contributed to unfavorable outcomes [20].

In addition to cancer-centered care, the pandemic has also affected the delivery of routine follow-up and laboratory visits. A considerable number of routine pediatric visits have been either postponed or canceled, leading to a decrease in subsequent referrals to specialists for lab results or symptoms that would have otherwise triggered a referral in the absence of COVID-19 [21]. Other patients with delayed diagnosis were noted to have prior contact with healthcare systems due to concern about abnormal symptomatology but were not referred to the emergency department or for further lab work, possibly due to the want to limit potential exposures to COVID-19 [6]. In a survey of U.S. parents conducted in March 2021 by Teasdale et al., 41% of U.S. children had missed a routine medical visit due to COVID-19 [21]. Delays in receiving routine healthcare could have long-term implications for the wellness of children. While the reluctance of parents to present children to care is an important contributor to reduced routine medical visits, the other side of the coin that also needs to be considered is the capability of healthcare centers to provide care.

A major setback faced by the healthcare sector during the pandemic is a significant staffing shortage. Fully saturated urgent care and emergency departments, as well as inadequacy in keeping up with the supply–demand chain by the providers, either due to a high volume of patients or a small number of providers because of illness, led to great difficulties in scheduling appointments [22].

A survey of worldwide healthcare providers conducted by Sniderman et al. revealed that COVID-19 had a profound effect on the physical, psychological, and financial well-being of pediatric healthcare providers [23]. This additional strain caused by COVID-19 on pediatric healthcare professionals also directly impacted pediatric oncology patients. Some of these hurdles faced by the healthcare system and providers were relieved by the utilization of alternative methods of providing care, such as telemedicine [6].

Telemedicine adoption among providers increased significantly during the pandemic. This has expanded the reach of healthcare services among the general population; however, it also has its own shortcomings. A study by Xie et al., reported that telemedicine was widely adopted in pediatrics during COVID-19; however, a deeper analysis of subspecialties revealed that pediatric oncology was one of the subspecialties that were categorized as low telemedicine adopters [24]. In the context of oncology, the lack of ability to detect critical physical exams findings such as unstable vital signs, pallor, and hepatosplenomegaly can be easily overlooked and missed in such virtual encounters [6]. Several countries noted their experiences with the COVID-19 pandemic. Norway noted a 50% increase in cancelations of pediatric hematology and oncology outpatient clinic consultation in March 2020 compared to 2019. They did, however, implement telehealth or telephone/video consultations to avoid delays in diagnosis [25].

Another factor contributing to the delay in diagnosis is the bias in diagnosis associated with COVID-19. Presenting signs and symptoms of cancer, such as fever and malaise, can be easily confused with COVID-19 symptoms given the overwhelming number of cases, thus creating a domino effect with untimely diagnosis and eventual poor prognosis [16].

**Figure 1:**
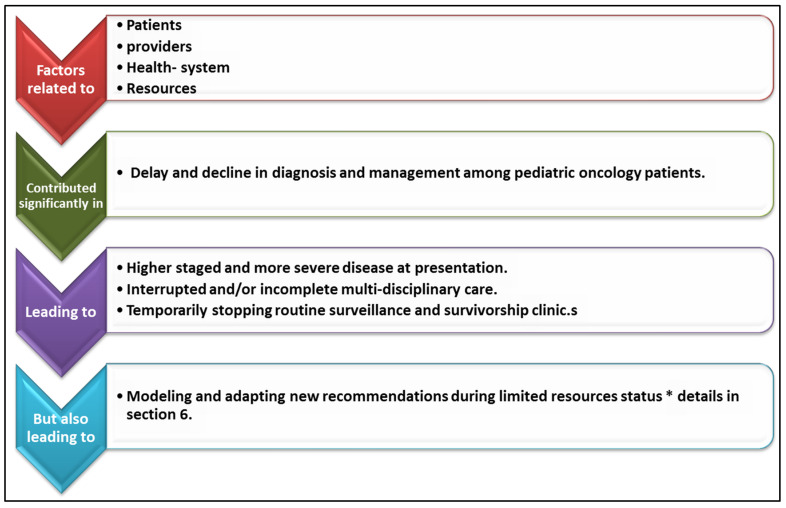
Schematic summarization of the effect of the COVID-19 pandemic on pediatric oncology diagnosis and management. * details in Section 6 entitled Management of the Malignancy during COVID-19.

In pediatric patients, new oncologic diagnoses require efforts made by a multidisciplinary team for appropriate evaluation and care. Workup typically includes imaging, biopsy, placement of central lines, initiating treatment, and referring to a center that specializes in this care. However, this may not always be the case, especially in countries around the world that do not have access to these resources of care. With the COVID-19 pandemic, there was an inability to obtain these resources or receive the appropriate quality of care and treatment globally but especially noted in resource-poor areas [26]. Reports in India suggested that with the COVID-19 pandemic and lockdown restrictions, there was a noted 50% decrease in new cancer cases being diagnosed. It was thought to be due to restrictions on travel implemented during the pandemic with limited access to seek medical care [12,13,14,26]. Hence, numerous patient-, provider-, and health-related factors have contributed significantly to the delay and decline in diagnosis among pediatric oncology patients during the COVID-19 pandemic. (Figure 1).

## 5. Increase Pediatric Leukemia “Occurrence” following SARS-CoV2 Infection and the Two-Hit Hypothesis

**Figure 2:**
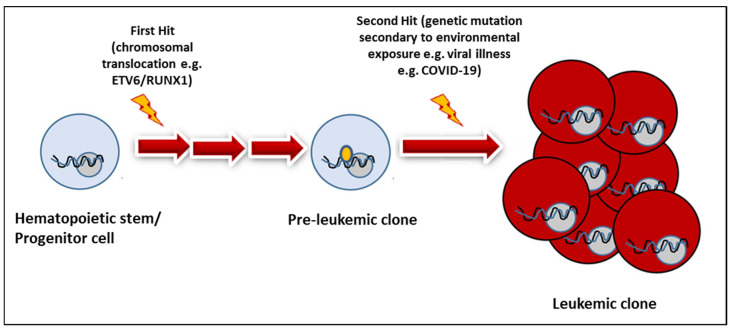
Demonstration of the two-hit hypothesis and COVID-19 can play a role in the second hit.

Children with malignancies constitute one of the most vulnerable subsets of the pediatric population. When COVID-19 first emerged, it stirred an interest among oncologic societies across the globe to study the impact this novel virus could have on various pediatric cancers. Dr. Greaves proposed the two-hit hypothesis for pediatric leukemogenesis. The first hit occurs prenatally with a noted preleukemic clone that may have a leukemia-associated genetic change, such as ETV6-RUNX fusion translocation. The second hit is exposure to an infectious agent in an already genetically susceptible child with preleukemic cells that may lead to an overstimulated immune response and allow for the proliferation of the leukemic cells [27,28,29]. Daycare attendance through the first 12 months of life has been shown to have a protective effect on ALL, as studied by the U.K. Children’s Cancer Study Group in the 1990s [30]. Further, consideration can be made that the closure of daycare centers and schools during the pandemic, as well as limited social interactions from the lockdown, may have caused the lack of immunological training for the patients [31]. This would describe the “second hit” in that these children had minimal infectious exposures throughout the first few years of their lives. However, once they became infected with a pathogen, their immune systems may have developed a heightened reaction that may have induced the development of leukemia [29,31]. (Figure 2).

Largeaud et al. were the first to demonstrate an interaction between lymphocytes and COVID-19 when they reported an increase in chronic lymphoid leukemia clones in a patient with COVID-19. This interaction was thought to be mediated via cytokine stimulation [32]. Thereafter, there have been a few reported cases of acute childhood leukemia post-COVID-19. Ioannidou et al. reported two cases of acute lymphoid leukemia (ALL) and one case of acute myeloid leukemia (AML) diagnosed in children in their institution after presenting with hematological abnormalities weeks to months after acute COVID-19 [33]. Another case of a 9-year-old child presenting with anorexia and bone pain a week after acute COVID-19 was later diagnosed as ALL [34]. No such causal association between COVID-19 and solid tumors or other childhood malignancies has been reported thus far. Thus, this emphasizes and supports the hypothesis of “second-hit” COVID-19 in the development of leukemia in pediatric patients.

## 6. Management of the Malignancy during COVID-19 (Figure 1)

Due to the COVID-19 pandemic, hospitals needed to change their practices due to surges causing overflow and limitations in resources. Oncology patients who typically received multimodal therapy and care had a limited ability to come to the hospital due to lockdowns in place. Early in the pandemic, leadership from the International Society for Pediatric Oncology (SIOP), Children’s Oncology Group (COG), SIOP-E (Europe), International Society of Pediatric Surgical Oncology (IPSO), Pediatric Radiation Oncology Society (PROS), Childhood Cancer International, (CCI), and St. Jude Global provided recommendations for the diagnosis and treatment of pediatric oncology patients. Care for pediatric oncology patients was not to be compromised due to the pandemic, and recommendations were made to establish normalcy during this time. They recommended that cancer centers have anticipatory and planned processes in the event hospitals became limited in resources, as evidenced in Lombardy, Italy [35]. Recommendations also came as an aid to low- and middle-income countries without adequate resources for the diagnosis and treatment of pediatric malignancies [36]. It was recommended to limit patient visits to clinics and hospital admissions and temporarily stop routine surveillance and survivorship clinics, especially in the event healthcare providers were needed in other departments for frontline critical care. Leadership had recommended that all aspects of cancer treatment would continue without any changes unless resources became limited [35,37].

Recommendations regarding anticipatory care in the setting of limited resources during the pandemic were modeled and adapted from the Lombardy experience in order to limit COVID exposures [35]. Factors included developing a standard operating procedure, COVID-19 viral testing of staff and patients prior to elective procedures or admission, appropriate hand washing and the use of appropriate personal protective equipment by staff and families entering and leaving clinical areas, restricting visitors to one per patient, cohorting staff, separation of oncology staff from staff working in COVID-19 areas, and an elective reduction of high-risk procedures, including CAR-T and stem cell therapies, to decrease the demand for intensive care services [35,37].

In certain instances where patients had advanced cancer with concurrent COVID-19, whether it is symptomatic or detected on screening, recommendations included interim therapy to control the disease and allow for recovery from COVID-19 before giving cancer-directed treatment. Similarly, in patients with new cancer diagnoses that were non-emergent, such as an abdominal mass or a low-stage Hodgkin’s lymphoma, deferring diagnostic approaches and treatment until recovery was deemed reasonable [37].

Leadership also provided recommendations in regard to ALL. It was recommended these patients undergo a full investigation to establish the diagnosis, risk stratification, and treatment plan according to guidelines, protocols, and trials. In patients who had COVID-19 alongside hyperleukocytosis, it was recommended that treatment include steroid prophase and supportive care and then initiating treatment-directed therapy once the patient had recovered from COVID-19 [38]. Typical diagnostics include age along with labs that include complete blood count, flow cytometry, molecular, bone marrow aspirate, and biopsy with cytomorphology to be interpreted. If flow cytometry and molecular diagnostics were unavailable, a diagnosis made without the use of these modalities was justifiable [39]. Patients would be treated with a morphologic response instead of molecular classification and minimal residual disease (MRD) status. No changes were recommended for maintenance therapy. Every attempt was to be made to minimize clinic visits in areas of high COVID-19 prevalence [37].

In regard to Burkitt Lymphoma, due to the aggressive nature of this disease, no modifications were recommended in fully resourced and high-income countries, especially in the setting of concurrent COVID-19. In settings where the disease was advanced with additional comorbidity, treatment prophase with stepped dosing corticosteroids and supportive care was recommended before initiating disease-directed therapy. In order to establish a diagnosis, baseline imaging such as chest X-ray, ultrasound, and biopsy were performed [40]. Hodgkin lymphoma recommendations included outpatient-based therapy without modifications in the protocol. Since low–middle-income countries may not have access to certain types of imaging for treatment stratification and radiotherapy access is limited, especially in the setting of COVID-19, a chemotherapy-only approach was recommended (in low- and intermediate-risk diseases) [41,42,43,44,45]. Several other modifications to the management of pediatric cancers were recommended [37].

It is also worth mentioning that, prior to major guidelines being released at the height of the pandemic, several countries released case reports of patients with altered chemotherapy regimens in the setting of COVID-19. Countries such as Japan, China, and Latin America had developed plans for AML with a milder treatment course [46,47]. Brazil followed their practices and treated nine patients with AML and COVID-19 with a less intensified induction regimen and showed excellent outcomes, with all achieving complete remission over the course of 4 months of follow-up [48]. Similarly, patients in China were followed over the course of 6 years and continued to have up to 4 years of follow-up at the time of the study completion. Their 4-year overall survival and event-free survival rate at 4 years were about 74% and 68%, respectively, with a cumulative incidence of relapse of about 25% [46]. A mild induction protocol regimen (MAG) consisted of mitoxantrone at 5 mg/m^2^ days 1, 3, and 5, cytarabine at 10 mg/m^2^ q12 for 10 days, and G-CSF 5 micrograms/kg once daily for 10 days [48,49]. Therefore, it was suggested that this regimen be used in low- and middle-income countries to overcome the induction mortality and try to improve outcomes of children and adolescents with AML, especially when in need of adapting a treatment regimen during an emerging health crisis when optimal therapy is not feasible. 

In September 2020, the countries in Africa took recommendations from the Lombardy region in Italy to utilize on their own pediatric oncology patients. There were 25 pediatric oncology centers from 15 countries in Africa that showed a decrease in pediatric oncology treatment activity due to family fear of COVID-19, restricted travel, decrease in parental finances [50]. It was noted that 60% of centers experienced staff shortages and had postponed surveillance consultations as well, leading to a decrease in activity in pediatric oncology treatment. It was also reported that modifications in chemotherapy administration, surgery, and radiation occurred due to drug shortages, delays of cancer surgeries, radiotherapy due to schedules, and a shortage of blood products to be given in 50% of participants. The countries also understood an anticipatory approach needs to be taken in that there may be an increased risk of receiving patients with advanced-stage cancer, risk of treatment abandonment and relapses, and to continue to be aware of this and prepare [50].

In India, 30 centers reviewed treatment modalities of malignancy during COVID-19, which included chemotherapy, surgery, radiotherapy, and hematopoietic stem cell transplant, and noted that 36% of patients experienced delays in their care. Some of these centers in India modified treatment protocols, increased growth factor uses, and increased the use of support from social organizations [12]. Seventy percent of treatment centers in India continued to treat malignancies such as ALL, AML, and sarcomas without any changes based on recommendations by the leadership [12].

The Pediatric Oncology East and Mediterranean (POEM) group reported on the impact of the COVID-19 pandemic on patients with established oncologic diagnoses. In the majority of centers surveyed, it was noted that off-therapy surveillance was delayed to decrease clinic load as was recommended by leadership groups [37,51]. There were noted to be delays in chemotherapy administration in 29% of the centers in the Middle East, North Africa, and West regions. Modalities for local control, such as surgery and radiation, were also delayed in 44% of centers, raising concerns about tumor recurrences and treatment failures [13]. Lebanon, for instance, delayed local control surgery and administered neoadjuvant chemotherapy in the midst of the pandemic to minimize the risk of infection [52]. Further, it was noted that acceptance of new pediatric oncology patients was restricted in 24% of the centers, with the potential for more patients being referred to other centers and possibly some patients unable to obtain the necessary access to curative therapy [13].

## 7. Management of COVID-19 in Pediatric Oncology

The National Institutes of Health (NIH) established guidelines regarding criteria for patients with COVID-19 based on clinical type. Data were limited for pediatric patients, given the small subset of patients affected by COVID-19, and guidelines were largely based on adult data. These guidelines would allow providers to decide between supportive therapy vs. antiviral treatment if needed in both non-hospitalized and hospitalized children (Table 1A–C) [53,54,55]. Symptomatology of COVID-19 included fever, cough, sore throat, malaise, headache, myalgias, nausea, vomiting, diarrhea, anosmia, and ageusia, among other symptoms, with or without supplemental oxygenation. Further guidelines were made regarding admitted patients with multisystem inflammatory syndrome in children (MIS-C), defined as a post-infectious complication of COVID-19 with symptoms including persistent fever, multisystem organ dysfunction, and systemic inflammation requiring care in the intensive care unit due to shock and the need for vasopressors and inotropes [56,57].

Initial evaluation for patients with suspected COVID-19 included chest imaging (X-ray, ultrasound, computed tomography) and an electrocardiogram. Laboratory tests recommended including a complete blood count with differential and a metabolic profile, including liver and renal function tests [53]. 

During the early phase of the pandemic, patients with a pediatric oncologic diagnosis needed to have a negative test by polymerase chain reaction (PCR) prior to the initiation of therapy. Poland has adopted European Centre for Disease Prevention and Control (ECDC) guidelines on ending COVID-19 isolation. In patients with mild–moderate disease, guidelines included no fever for 3 days and clinical improvement of symptoms as well as either 10 days having passed since isolation, or two negative COVID-19 PCR tests spaced 24 h apart [58]. In patients with severe disease, similar criteria were applied with the modification of a minimum of 14 days, and up to 20 days have passed since isolation. Similarly, in immunocompromised patients, a modification of 20 days needed to pass after the onset of symptoms was applied in order to leave isolation [58,59]. As these were guidelines developed earlier in the pandemic and with more knowledge and awareness of how COVID-19 affects patients, it is now recommended to begin treatment as soon as feasible, as delaying the induction phase could have an adverse effect on the outcome of the patient [60]. Further reports have also suggested not delaying treatment, especially as leukemia is curable, and it is noted that pediatric patients typically have a less severe infectious course with COVID-19 [61] and have shown success in treatment when receiving concurrent care with an active infection from COVID-19 [62].

The COVID-19 pandemic further affected procedures and surgeries that would take place for patients with malignancies. Institutions largely required patients to have a negative COVID-19 test prior to each sedated procedure. Positive test results potentially caused cancelations or delays in procedures until consecutive results yielded negative results [63]. 

Prior to a consensus, there were recommendations on the prevention or management of pediatric oncology patients in the setting of COVID-19. Recommendations include the patient being isolated to their home in between receiving treatment, and if they are to receive treatment, they stay in a single and nonshared room. Outpatient follow-up appointments were limited to an urgent basis, and there was an increase in the use of telemedicine or other communicative efforts [51]. COVID-19 screening of symptomatic patients was initiated, social distancing rules of at least 6 feet were enforced between individuals with no grouping, and a one-parent limit to stay with the child was recommended inpatient and outpatient. Recommended that, if possible, hospital settings could establish a COVID-19-free site to provide scheduled treatment [51]. Along the same lines, an inpatient setting dedicated just to COVID-19 pediatric oncology patients was trialed in Italy in order for patients who were infected by the virus to still continue their treatment under adequate medical and nursing assistance [64].

Limited data had previously suggested that patients with cancer and COVID-19 may have an increased risk of complications and mortality in comparison to the general population [65]. Lee et al. observed that patients with hematological malignancies were at increased risk of COVID-19 compared to those with solid tumors and exhibited a more severe clinical course [60]. However, as time passed, it was learned that children with malignancies were not necessarily at increased risk of severe COVID-19 [66], though symptomatic management was recommended in this high-risk group of patients. It is unclear about pediatric oncology patients’ increased risk for complications and mortality when there is associated COVID-19. Treatments have included the use of remdesivir due to in vitro activity against COVID-19 [65]. A first case report in the U.K. presented a pediatric patient with acute leukemia and COVID-19 who received concurrent treatment with remdesivir and induction chemotherapy and was shown to have not deteriorated or suffered severe complications from COVID-19 and was also in remission at the end of induction. This patient had a high risk of immunosuppression due to his chemotherapy regimen and was also at risk of worsening viral infection but was able to tolerate these regimens well [65]. Further cases revealed similar reports of patients with acute leukemia along with COVID-19 who were started on antiviral therapy following infection with COVID-19 and had successful outcomes [67,68].

Finally, in the setting of the COVID-19 pandemic, vaccines were developed to help prevent infection. AIEOP formulated a consensus report consisting of 21 statements and their rationale to specifically address the need for vaccination in this vulnerable immunocompromised population of pediatric oncology patients. It specifically addressed the questions regarding the need for vaccination, type and schedule, timing of vaccination, and eligibility criteria, among many other criteria that have been discussed in detail in this consensus report [69]. COVID-19 vaccines are now recommended in all patients older than 6 months who are undergoing chemotherapy and after a hematopoietic stem cell transplant. This is recommended to reduce the risk of therapy interruptions and complications that can occur from COVID-19 [70]. It is recommended to give vaccination at least 14 days prior to starting therapy treatment, but if this is not possible, then it is recommended to administer the vaccine when cytopenia from treatment is minimal. In bone marrow transplant patients, vaccines are recommended as soon as 100 days after the transplant and CAR-T cell therapy [71]. Once these patients have completed therapy, another dose is indicated to increase the immune response, as well as a booster dose every few months to maintain immunity [71]. The vaccine is noted to be safe and effective in children, especially those who are immunocompromised [72]. It is of note that the efficacy of the vaccine is lower in immunocompromised patients, and there have been no studies to date measuring vaccination efficacy in children with cancer [72,73], making it of utmost importance to continue taking protective measures such as vaccination.

## 8. Conclusions

This review demonstrated the impact COVID-19 had on the pediatric oncology community. There were several factors that needed to be considered prior to the initiation of oncologic management of pediatric patients to achieve the best results. Although studies largely indicated COVID-19 was not as severe in pediatric patients, it remained a concern, especially in the setting of pediatric patients with malignancies. Several guidelines have been formulated by global oncologic societies for the management of pediatric malignancies in the setting of the COVID-19 pandemic and especially in the setting of concurrent infection. As COVID-19 remains a concern today, it will be interesting to see what else will be learned about this infection in the near future.

## Figures and Tables

**Table 1:** (**A**) Recommended COVID-19 management in non-hospitalized children [53,54]; (**B**) recommended COVID-19 management in hospitalized children [53,55]; (**C**) recommended MIS-C management in hospitalized children [51,53,55,56].

(A)				
Clinical Type	Acute			
COVID-19 Disease Severity	Symptomatic with or Without Risk Factors	Low	Intermediate	High
Comorbidities	None	Mild asthma, overweight, well-controlled diabetes mellitus	Sickle cell disease, poorly controlled diabetes mellitus, non-severe neurologic, metabolic, or cardiac disease, ≤2-year-old children born premature, age <1 year	Moderate to severely immunocompromised, obesity, severe underlying respiratory disease, severe congenital or acquired cardiac disease, or severe neurologic, genetic, metabolic disability with impaired airway clearance
Associated risk of progression to Severe COVID-19		Weak/unknown	Moderate or inconsistent	Strong or consistent
Recommended Treatment	Supportive care	All ages: Supportive care	All ages: Insufficient evidence for or against antiviral therapy	Age <12: Insufficient evidence for or against antiviral therapy Age 12–17: remdesivir within 7 days or symptoms or ritonavir-boosted nirmatrelvir (paxlovid) within 5 days of symptoms **
**(B)**				
**Severity of disease**		**Recommendations**		
Hospitalized for COVID-19		Prophylactic anticoagulation unless otherwise indicated in children >/12 years		
No O_2_ supplementation		Consider remdesivir in children with high-risk disease severity ages 12–17. Insufficient evidence in children <12 years		
O_2_ supplementation (not high flow, noninvasive, mechanical ventilation, or ECMO)		Remdesivir or dexamethasone plus remdesivir for children with increased oxygen demands		
O_2_ supplementation (high flow, noninvasive ventilation)		Dexamethasone or dexamethasone plus remdesivir If no improved oxygenation, consider baricitinib or tocilizumab ages 2–17		
Requiring mechanical ventilation or ECMO		Dexamethasone If no improved oxygenation, consider baricitinib or tocilizumab ages 2–17		
**(C)**				
**Recommended treatment**		**Instructions**		
Initial immunomodulatory therapy		IVIG 2 g/kg ideal body weight/dose IV plus methylprednisolone IV (low to moderate dose 1–2 mg/kg/day) or equivalent glucocorticoid		
Refractory immunomodulatory therapy (no improvement within 24 h of initial therapy)		Start 1 of the following: High dose anakinra IV or subq daily (5–10 mg/kg) Methylprednisolone IV (higher dose 10–30 mg/kg/day) or equivalent glucocorticoid Infliximab 5–10 mg/kg IV for 1 dose		
Antithrombotic treatment		Low dose aspirin PO (3–5 mg/kg/day with maximum 81 mg) for children without risk factors for bleeding AND Therapeutic anticoagulation dependent on moderate to severe left ventricle dysfunction, large coronary artery aneurysm, or consideration of prophylactic/therapeutic anticoagulation without above symptoms		

** Ultimately, admission would be required in this risk group.

## Data Availability

Not applicable.
